# Mucocele-like lesions of the breast: a long-term follow-up study

**DOI:** 10.1186/1746-1596-6-29

**Published:** 2011-04-06

**Authors:** Yasuyo Ohi, Yoshihisa Umekita, Yoshiaki Rai, Yasuaki Sagara, Shinichi Baba, Shugo Tamada, Yoshiaki Sagara, Taeko Kukita, Takako Yoshioka, Kazuhito Hatanaka, Yoshiatsu Sagara, Akihide Tanimoto

**Affiliations:** 1 Hakuaikai Sagara Hospital, 3-31, Matsubara, Kagoshima 892-0833, Japan; 2 Department of Molecular and Cellular Pathology, Field of Oncology, Kagoshima University Graduate School of Medical and Dental Sciences, 8-35-1, Sakuragaoka, Kagoshima 890-8544, Japan

## Abstract

**Background:**

Mucocele-like lesions (MLL) of the breast were originally described as benign lesions composed of multiple cysts lined by uniform flat to cuboidal epithelium with extravasated mucin, but subsequent reports described the coexistence of columnar cell lesions (CCL), atypical ductal hyperplasia (ADH) and ductal carcinoma in situ (DCIS). Several reports have investigated whether core biopsy can diagnose MLL reliably; however, there is only one report with a long-term follow-up after excision of MLL. We report here 15 surgically excised MLL with a long-term follow-up.

**Findings:**

Fifteen lesions diagnosed as MLL from 13 patients who had undergone excisional biopsy between January 2001 and December 2006 were retrieved and followed-up for 24-99 months (median 63.8). Two lesions were accompanied with CCL, 5 with ADH and 3 with low grade DCIS. Four lesions (2 ADH, 2 DCIS) were additionally resected and their histology revealed 2 ADH, one DCIS and one MLL with CCL. Of 4 lesions (3 ADH, one DCIS) without additional resection, one lesion (ADH) relapsed accompanied with DCIS at 37 months after excision.

**Conclusions:**

MLL were frequently accompanied with CCL, ADH or low grade DCIS. Complete resection may be recommended in case of MLL with ADH or DCIS because of intralesional heterogeneity and the probabilities of relapse.

## Introduction

Mucocele-like lesions (MLL) of the breast were first described by Rosen as benign lesions analogous to mucoceles of the minor salivary glands [1]. MLL was defined as mucin-containing cysts lined by flat or low-cuboidal epithelium, with extravasated mucin into the surrounding stroma. The term MLL does not imply the nature of the accompanying epithelial alterations, which ranges from benign changes to columnar cell lesions (CCL), atypical ductal hyperplasia (ADH), ductal carcinoma in situ (DCIS) or mucinous carcinoma [2-5]. Although the main focus of the study, to date, has been whether core biopsy reliably can diagnose MLL [5-8], there is only one report with a long-term follow-up after excision of MLL [2]. We report here a long-term follow-up study of 15 surgically excised MLL.

## Materials and methods

We analyzed 15 lesions diagnosed as MLL from 13 patients who had undergone an excisional biopsy at Hakuaikai Sagara Hospital (Kagoshima, Japan) between January 2001 and December 2006. Thirteen lesions were detected with calcification by mammography and one lesion by ultrasound examination. One lesion showed a palpable mass. All patients were female and the mean age was 48.9 years (range: 38-56). The patients were followed-up biannually for 24-99 months (median: 63.8 months) by palpation, mammography and ultrasound examination. CT examination was additionally performed for cases with DCIS. CCL was diagnosed according to the classification system outlined by Schnitt and Vincent-Salmon [9]. ADH, DCIS and lobular neoplasia were diagnosed using established criteria according to the WHO classification [10]. The surgical margin of excisional biopsy specimens was histologically examined in all directions. Dilated mammary ducts containing mucus were regarded as a continuity of MLL. The surgical margin was defined as the area within 3 mm from the edge. Histological evaluation was performed by two pathologists (YO and YU) and a final agreement was reached using a discussion microscope when the assessment differed.

## Results

**Figure 1:**
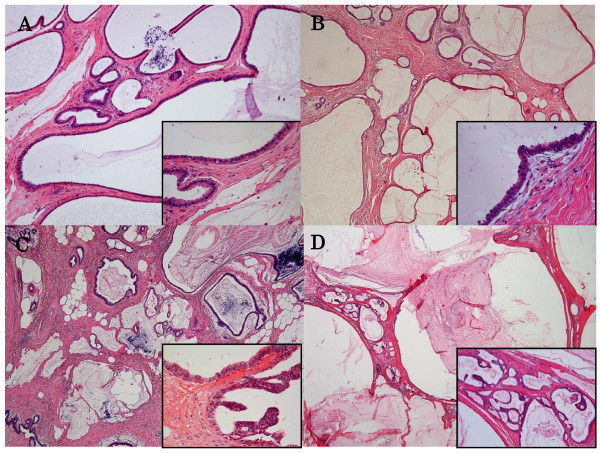
**Mucocele-like lesions without (A) or with columnar cell lesions (B). Mucocele-like lesions with atypical ductal hyperplasia (C) or ductal carcinoma in situ (D)**. A. Numerous cysts containing mucinous secretion are lined by a thin epithelial layer accompanied with extravasated mucin. Cytological atypia is not evident (inset) (H.E.). B. Besides histology of mucocele-like lesion, the stratification of epithelium is noted in the lining epithelium (inset) (H.E.). C. Micropapillary pattern is associated with atypical lining epithelium (H.E.). D. Micropapillary and cribriform pattern are noted (H.E.).

Formalin-fixed paraffin-embedded sections were made from the entire excisional biopsy samples by slicing at 3 mm intervals. All lesions showed distended mucin-filled ducts lined by almost entirely single layer cells with mucin extrusion into the stroma. The histology, treatment and prognosis are summarized in Table 1. Two lesions were diagnosed as MLL with CCL. Representative figures of MLL without CCL and MLL with CCL are shown in Figure 1A and 1B, respectively. Case 1 had two different breast lesions: one in the left upper outer quadrant which was diagnosed at the age of 52, and another in the left upper inner quadrant which was diagnosed at the age of 55. Case 6 was associated with lobular neoplasia near the MLL. Case 11 showed bilateral breast lesions at age of 49 years. Five lesions were associated with ADH (Figure 1C) and 3 lesions with DCIS. All cases of DCIS consisted of low grade micropapillary and/or cribriform patterns (Figure 1D). In 7 lesions (4 MLL with ADH, 3 MLL with CCL) at the surgical margin, additional resection was performed in 4 lesions (case 10, wide excision; case 11 and 12, mastectomy). Case 10 revealed the same histology as was seen at the excisional biopsy. The right breast lesion in case 11 showed MLL with DCIS at additional resection, while the left lesion showed MLL with ADH. The left lesion was accompanied with DCIS in a different quadrant breast area from the MLL at additional resection. Case 12, diagnosed as MLL with DCIS at excisional biopsy, showed MLL without CCL at additional resection. Only case 9 relapsed 37 months after excisional biopsy associated with increasing calcification during the follow-up period. The additional resection performed in case 9 revealed MLL with DCIS. Subsequently, all patients have been relapse-free and neither lymph node nor distant metastasis has been detected.

**Table 1:** Histology, treatment and prognosis in fifteen mucocele-like lesions

**Case**	**Age** **years**	**Site**	**Diagnosis at excisional biopsy**	Histology at additional resection*	**RFS** **(months)**	**OS** **(months)**
1	52	L	MLL without CCL	not performed	99	99
1	55	L	MLL with ADH	not performed	63	63
2	52	R	MLL without CCL	not performed	71	71
3	47	L	MLL without CCL	not performed	24	24
4	51	R	MLL without CCL	not performed	90	90
5	53	L	MLL without CCL	not performed	55	55
6	45	L	MLL with CCL	not performed	52	52
7	40	R	MLL with CCL	not performed	70	70
8	56	L	MLL with ADH	not performed	62	62
9	44	L	MLL with ADH	not performed	37 (relapsed)	88
10	46	L	MLL with ADH	MLL with ADH	52	52
11	49	R	MLL with ADH	MLL with DCIS	51	51
11	49	L	MLL with DCIS	MLL with ADH	54	54
12	38	R	MLL with DCIS	MLL without CCL	82	82
13	56	R	MLL with DCIS	not performed	44	44

MLL: mucocele-like lesions, ADH: atypical ductal hyperplasia, CCL: columnar cell lesions, DCIS: ductal carcinoma in situ,

RFS: relapse-free survival, OS: overall survival

*mastectomy (case 11, 12), wide excision (case 10)

## Discussion

MLL of the breast was originally described by Rosen as benign lesions composed of multiple cysts lined by cytologically uniform flat or cuboidal to columnar epithelium with extravasated mucin [1]. Subsequent reports have shown an association with CCL, ADH, DCIS and mucinous carcinoma and suggested that MLL and mucinous carcinoma may represent two ends of the pathological spectrum of mucinous lesions of the breast [2-5]. CCL were defined as enlarged terminal duct lobular units with variably dilated acini, secretion, apical snouts and varying degree of cytological atypia [9], and a frequent association with MLL has been reported [5]. Excisional biopsy has been recommended for MLL diagnosed by core biopsy when mucin-filled ducts or cysts and stromal mucin are found, particularly if a mass is evident radiologically or by palpation, or if the sample exhibits ADH [6,7]. On the other hand, Wang *et al. *[8] reported that the diagnosis of MLL without atypia by core biopsy may not require surgical excision. Begum *et al. *[5] also stated that surgery may be avoided in benign MLL when the entire radiological abnormality has been removed by large core mammotome biopsy. To our knowledge, there is only one report with a long-term follow-up after excision in 49 patients with MLL, in which two patients had local recurrences [2]. In one of these cases, the initial tumor was MLL with ductal hyperplasia, and MLL with lobular carcinoma in situ recurred 2 years after excisional biopsy. In the other case, benign MLL relapsed 5 years after excisional biopsy, diagnosed as benign MLL. In the present study, 53% (8 of 15) of MLL were accompanied with ADH or low grade DCIS.

Complete resection may be desirable in case of MLL with ADH or DCIS because of intralesional heterogeneity and the probabilities of relapse.

## Competing interests

The authors declare that they have no competing interests.

## Authors' contributions

YO designed the study, performed the histopathological examination and wrote the manuscript. YU designed the study, interpreted the data and wrote the manuscript. YR, YS, SB, ST, YS, TK and YS participated in the sampling of clinical data. TY, KH and AT critically read the manuscript and supervised. All authors read and approved the final manuscript.
